# Frailty Related to Anesthesia guided by the Index “bispectraL” (FRAIL) study: study protocol for a randomized controlled trial

**DOI:** 10.1186/s13063-017-1868-9

**Published:** 2017-03-16

**Authors:** Morgan Le Guen, Marie Herr, Antoine Bizard, Caroline Bichon, Nathalie Boichut, Thierry Chazot, Ngai Liu, Joel Ankri, Marc Fischler

**Affiliations:** 1 Department of Anesthesiology, Hôpital Foch, University Versailles-Saint Quentin, 40 rue Worth, 92150 Suresnes, France; 2 UVSQ, UMR-S 1168, Université Versailles Saint-Quentin-en-Yvelines, Saint-Quentin-en-Yvelines, France; 3 INSERM, U1168 VIMA: Aging and chronic diseases. Epidemiological and public health approaches, Villejuif, France; 4 Département de Santé Publique, AP-HP, Hôpital Sainte Périne, Paris, France; 5 Department of Neuropsychology and Mobile Unit for Geriatric Patients, Hôpital Foch, University Versailles-Saint Quentin, Suresnes, France; 6 Department of Anesthesiology and Critical Care Medicine, CHRU Besançon, University of Franche-Comté, Besançon, France

**Keywords:** Frailty, Elderly, Anesthesia, Outcome, Recovery

## Abstract

**Background:**

Currently, patients older than 60 years of age represent 25% of the population and are at an increased risk during surgery. Therefore, reducing postoperative morbidity and mortality is a major concern in medical research and practice. Dependence on caregivers and cognitive impairment represent two major risk factors in the elderly, especially in frail patients after surgery under general anesthesia. In this context, continuous monitoring of the depth of anesthesia using a bispectral index (BIS) sensor may reduce the occurrence of impairments by gaining better control of the anesthetic depth. The first aim of this study is to compare manual versus automated administration of intravenous anesthetics with regard to 6-month functional decline in persons aged 70 years and older. The secondary objective includes an evaluation of the influence of the frail phenotype on self-sufficiency in elderly patients after general anesthesia.

**Methods/design:**

After receiving ethical committee approval and written consent, a complete preoperative assessment of physiological reserve and self-sufficiency will be performed on patients more than 70 years old who are scheduled for surgery under general anesthesia. This evaluation will determine the patient’s frailty status in three categories: robust, pre-frail, and frail. Then, patients will be randomized into two groups: manual administration of anesthetics guided by BIS sensor (manual group) or automated administration (automated group) with recording of the anesthesia. A second examination will be scheduled after 6 months to assess changes in functional abilities, cognitive functions, and frailty status. A priori calculation of sample size gives a population of 430 patients to be included in this multicenter trial.

**Discussion:**

This clinical study is designed to detect any postoperative complications and deaths related to the performance of the general anesthesia guided by the BIS sensor and the preoperative functional status of the elderly: robust, pre-frail, or frail.

**Trial registration:**

ClinicalTrials.gov, NCT02524327. Registered on 10 August 2015.

**Electronic supplementary material:**

The online version of this article (doi:10.1186/s13063-017-1868-9) contains supplementary material, which is available to authorized users.

## Background

In 2010, 16.7% of the French population was over the age of 65, with that number projected to rise to 20% in 2020 according to the National Institute of Statistics and Economic Studies (INSEE). Indeed, this trend is of concern in every developed country. Moreover, progress in surgical techniques and control of anesthesia means that surgical procedures are becoming more common in the elderly, with approximately 30% of surgeries conducted in patients over the age of 70, according to the latest epidemiological surveys. However, aging is accompanied by certain physiological changes that may have a critical impact on the patient’s postoperative outcome, including reduced adaptation in cardiac output to stress, reduced lung compliance, decreased cerebral blood flow, thermal dysregulation, loss of functional units in the kidneys, and decrease in renal blood flow [1].

Increasing age in the general population has led to the development of the concept of frailty to better assess the geriatric patient population [2]. Frailty represents a decrease in homeostasis and resistance to stress, and it increases the vulnerability of a person exposed to the risk of unfavorable changes (morbidity or death) disproportionately in relation to the event [2, 3]. Physiologically, fragility results from the simultaneous impairment or decrease in physiological reserve in multiple systems. The early detection of at-risk individuals is accomplished through geriatric assessments using psychometric scales and the Fried score, which is the most widely used metric scale for frailty and includes five criteria for determining anesthetic or surgical perioperative risk factors. This type of assessment attempts to prevent functional decline and high morbidity and mortality in elderly surgical patients [4, 5]. In this specific situation, a relationship between depth of anesthesia and impaired postoperative outcome has been described in a large but retrospective cohort. This suggested a more pronounced impact on the elderly and especially in pre-frail or frail patients.

The anesthetic risk is increased in the elderly, and a previous survey demonstrated that mortality per 100,000 patients under anesthesia reached 21 in patients 75 years and older, while it was only 5.2/100,000 in patients aged between 45 and 74 years [6]. Considering that anesthesia guided by a bispectral index (BIS) sensor allows the depth of anesthesia to be standardized regardless of comorbidity, the variability of BIS values (oscillations, low values, and the presence of burst suppression) could evoke a window to the brain metabolism and an imbalance between needs and posology. These situations suggesting a possible overdose in anesthetics could have a negative impact on cognitive functions and subsequent postoperative morbidity and mortality. Therefore, identification of these risk factors is essential to provide suitable anesthetic management during surgery. Thus, one of the first lines of therapeutic actions could be the wide use of vasopressors to limit cerebral hypoperfusion and thereby reduce exposure to burst suppression. Another possibility is the use of an automated controller of anesthesia. Our team has previously demonstrated the superiority of a controller versus human-guided anesthesia in maintaining a target of BIS between 40 and 60 while limiting the incidence of too deep anesthesia, which is often associated with the occurrence of a suppression ratio in the elderly [7].

Our hypothesis is that accurate control of the anesthetic depth to limit episodes of too deep anesthesia and burst suppression (isoelectric electroencephalogram (EEG)) could decrease mortality and the occurrence of frailty with a loss of autonomy in the elderly. In the current literature, the absence of such a control leads to a mortality rate of 10% at 6 months in patients more than 70 years old, while the loss of functional capacity approaches 20%, meaning that approximately one third of elderly patients are at risk of experiencing a major negative impact following general anesthesia.

## Methods/design

This study is a multicenter, randomized, single-blinded trial performed with two parallel arms. The trial is recorded as NCT02524327 (available on the website: https://clinicaltrials.gov/ct2/show/NCT02524327), and it received ethical approval from the local committee.

### Eligibility and inclusion

The inclusion criteria included American Society of Anesthesiologists (ASA) grades I–III adults of both sexes more than 70 years old who were scheduled for surgery lasting up to 60 min, living at home or living in institutions without daily medical care, and able to be contacted by phone during the follow-up period. The exclusion criteria included having been diagnosed with dementia or having a severely impaired psychometric test (Mini-Mental State Examination (MMSE) score <15), any disability that would make the geriatric assessment incomplete (visual, auditory, or disabling apraxia), severe brain pathology (tumor, stroke, Parkinson's disease, etc.), or patients with related contraindications for BIS monitoring (pacemaker, brain surgery, cardiac surgery, or any surgery that prevents the analysis of the BIS under suitable conditions).

### Frailty status

Although it does not appear systematically during aging, which suggests a genetic component and a role for environmental factors [8], the prevalence of frailty increases with age and ranges from 4% in subjects aged 65–69 years to 26% in subjects older than 85 years. Collard et al. estimated that frailty concerns 10% of people over the age of 65 [9]. Although the concept of frailty is now recognized, there is no consensual definition or screening tool for frailty. Instead, frailty is observed as an accumulation of deficits [10] or as an independent phenotype [4]. In both operational approaches, frailty is defined by the risk of death, functional disability, or institutionalization. Central to this model is the occurrence of sarcopenia, or muscle loss related to age, which is often caused by undernutrition. In this study, we have chosen the model of the frailty phenotype (Fried's scale) resulting from the US Cardiovascular Health Study (CHS), which describes the five operational criteria of frailty. To date, this scale is the most common assessment used in the literature because it allows a quick assessment of an elderly patient by any healthcare provider. The scale is based on the following assessments:

1. Exhaustion. Exhaustion is classified using the Center for Epidemiologic Studies Depression Scale (CES-D) indicator: *I felt that everything I did was an effort; and I could not get going* with the following question: *How often in the last week did you feel this way?* The responses are rated as 0/rarely or none of the time (1 day), 1/some or a little of the time (1–2 days), 2/a moderate amount of the time (3–4 days), or 3/most of the time. Specifically, 1 point is given for subjects answering “2” or “3” to either of these questions.
2. Resistance or aerobic activity (level of physical activity). The quantitative assessment of the energetic expense is performed by using the International Physical Activity Questionnaire-Elderly (IPAQ-E), which distinguishes three levels of activity (low, moderate, and high) according to time spent walking and performing moderate (e.g., carrying light loads, leisurely bicycle rides, or tennis) and vigorous activity (e.g., carrying heavy loads, digging, lifting a pack of six bottles, or speed bicycling) during the past 7 days [11]. Specifically, 1 point is given for low levels of activity.
3. Unintentional loss of weight. Specifically, 1 point is given for a positive response to the question: *Have you lost more than 5 kg in the past year?*
4. Slow walking. This is measured by determining the time it takes for a patient to walk 4 meters (threshold according to gender and height). Specifically, 1 point is given to any patient with a slow walk.
5. Muscle weakness. This is assessed by evaluating hand grip strength (threshold according to gender and body mass imdex (BMI)). Specifically, 1 point is given if the patient scores below the reference.

At the end of this assessment, a subject is classified as "frail" if the total criteria total more than 3. Subjects with a criterion of 1 or 2 are referred to as "pre-frail," and subjects with no criteria are considered "robust."

### Conduct of the study

**Fig. 1:**
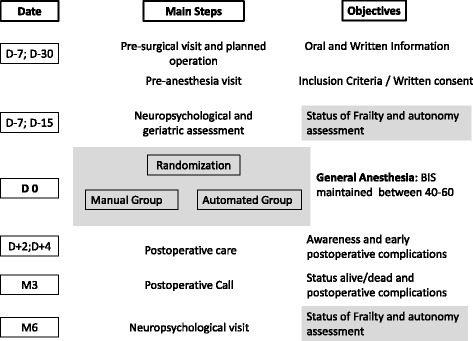
Flowchart of the study setup

**Fig. 2:**
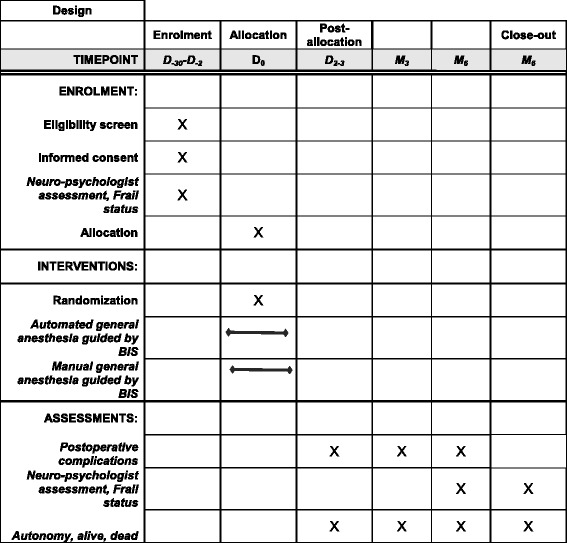
Example template of recommended content for the schedule of enrollment, interventions, and assessments

Figures 1 and 2 show, respectively, a flowchart of the study setup and the Standard Protocol Items: Recommendations for Interventional Trials (SPIRIT) template. See Additional file 1 for the SPIRIT checklist.

#### Presurgical period

After surgical consultation, the patients will receive written information about the study. Time will be devoted to responding to the patient’s questions before collecting the patient’s consent during the pre-anesthesia visit, which is used to confirm that the patient meets the inclusion criteria. If he/she agrees to participate in the research, this consultation will be immediately followed by a specialized consultation with a neuropsychologist trained to perform an assessment of functional decline and frailty in addition to a cognitive assessment. This visit is designed based on the experience of the epidemiology and geriatric units. The number of items in the questionnaire is limited to avoid fatigue in the participants, especially since the visit includes a measure of physical performance.

#### Randomization

On day 0, upon arrival at the operating room, centralized randomization on a website will be performed with stratification of the frailty status prior to surgery (phenotypic Fried scale in three categories: robust, pre-frail, frail) crossed with two categories of surgical risk: minor or major. This randomization will determine the modality of total intravenous anesthesia (manual or automated group). For the first arm, total intravenous anesthesia is achieved under the exclusive direction of an anesthesiologist with a BIS value maintained in the range 40–60 as the target — *the manual group* (this is the usual technique used for intravenous drugs). The BIS is an absolute value from 0 to 100 derived from the frontal EEG: 100 corresponding to a fully awake patient and 0 to a flat EEG. The second arm involves completely automated delivery; a proportional-integrative-differential (PID) algorithm is used to manage the administration of both propofol and remifentanil through a controller to reach a BIS target in the range 40–60 — *the automated group*. The PID algorithm means that the controller calculates the difference between the measured output and the setpoint (BIS = 50) to adjust the input value. The algorithm considers the present (proportional), past (integral), and future (derivative) error to adjust for the correction. At any moment, the automatic control can be over-ridden by the physician. This event is recorded for further analysis.

#### Anesthesia management

For anesthesia management, typical monitoring (electrocardiogram (EKG), pulse oximetry, noninvasive blood pressure) or invasive monitoring (neuromuscular blockade, capnography, inspiratory oxygen fraction) will be set up for the patient with mandatory BIS monitoring. Intubation or the establishment of a supraglottic device will be at the discretion of the anesthesiologist, as well as the use and selection of a muscle relaxant. Fluid management and hemodynamic control are left to the practitioner’s appreciation. Any perioperative events such as prolonged arterial hypotension, sudden bleeding, and myocardial ischemia may indirectly impair the patient's prognosis. Therefore, to limit any bias in the analysis, some targets were common among the investigators: limit any episode of arterial hypotension (meaning a drop >20% compared to baseline), avoid anemia (maintain hemoglobin >8 g/dl in absence of ischemic event), perform ST-segment monitoring, etc. All events are systematically recorded during anesthesia [12]*.* Prophylaxis against postoperative nausea and vomiting is administered according to the Apfel score [13]. Rapid tube removal after stopping anesthesia is strongly recommended to avoid any prolonged sedation in this cohort of patients. The investigator is not blinded to the anesthesia technique (control or automated), but he does not know the frailty status; likewise, the controller in the automated group does not know it. The patient and the neuropsychologist performing the postoperative assessment are blinded, and this decreases the risk of bias.

#### Follow-up

Postoperative care will follow the rules of standard care with an additional interview conducted between days 1–3 to ensure the absence of any complications and awareness. Leclerc [14] has previously described the questionnaire used to assess explicit memory. During the third month, a healthcare professional will telephone to determine the patient’s condition upon returning home (e.g., delayed complications, delirium, and need for a transitional care unit) and will seek to determine if any limitations in activity exist or, at the worst, if the patient has died. Finally, during the sixth month, a new specialized consultation will be planned with the repeated application of psychometric tests blinded to the mode of anesthesia used. The study ends with this consultation. If the patient refuses or is unable to come to the second visit, a telephone interview will be planned to assess his/her health, functional, frailty, and cognitive status. Regarding health and functional abilities, the same questions as those included in the questionnaire will be asked during the telephone interview. Cognitive functions will be examined using the screening tools that were validated by telephone: the F-TICSm questionnaire (French adaptation of a version of the phone MMSE) and the Category Fluency by Telephone (CFT) [15]. Frailty will be assessed by asking the participants about unintentional weight loss, fatigue, and physical activity. The measure of muscle weakness and slow walking speed will be approximated by any difficulties lifting a 5-kg bag and difficulties climbing up and down stairs.

Likewise, the following events will be documented: any event occurring between neuropsychological assessment (inclusion visit) and surgery (randomization) that is considered important by the physician anesthesiologist managing the patient and that may change the status of the patient and may lead to the patient being excluded from the study or declared as an undesirable effect in case of return to hospital, or any other surgery or medical event during this interval, or death.

### Data collected

#### Demographic data

Typical demographic data, such as age, sex, BMI, and ASA status, will be collected. Comorbidity using the Charlson index will be recorded to correlate with the follow-up results. Finally, a specific investigation regarding surgery or a fall during the previous year will be performed. This section also includes a global health assessment to detect any symptoms of depression and any signs of social isolation.

#### Functional abilities

Any limitations in activity will be evaluated using the six activities of daily living (ADL) included in the Katz index [16, 17], which consist of eating, bathing, dressing, toileting, transferring (walking), and continence, as well as instrumental activities of daily living (IADL) [18], such as food preparation, the ability to use a telephone, housekeeping, shopping, and the ability to manage one’s finances.

#### Cognition

Cognitive function will be assessed using different tests. First, the MMSE will be used to confirm the absence of severe cognitive impairment with a score above 15. Nevertheless, if the patient remains self-sufficient, this is not an exclusion criterion. Then additional tests, such as the Trail Making Test, word recall, verbal fluency, and drawing a clock at 10:10, are performed.

#### Frailty

Frailty as a phenotype was calculated using Fried's assessment as previously described. However, Edmonton's criteria will also be collected because they explore other dimensions such as social aspects or the patient's medication. Finally, the Timed Up and Go test was added to explore proprioceptive dimensions.

#### Collection of data

During every visit, a case report form (CRF) — created after the written consent is obtained — with an anonymous number is filled out by any investigator involved (doctor, neuropsychologist) from the inclusion to the last interview with the neuropsychologist at the sixth month. In case of a major undesirable event, the number can be unmasked to inform or find the patient if any residual risk exists. The data monitoring in this study will be locally managed without any sponsor by our clinical research team (DRCI Hôpital Foch, URC Besançon), and the final validation will be performed by a specialized society with no competing interest in this study. There are no financial or other competing interests for principal investigators for the overall trial and each study site. The study is supported only by a grant from the French Health Ministry.

### Statistical analysis

The main outcome will be defined as "the percentage of patients alive without loss of autonomy in the sixth month (26 ± 2 weeks) following surgery." By definition, death will be included as a permanent loss of autonomy of the subject. Secondary outcomes include a comparison of the postoperative complications using Dindo and Clavien's classification, a change in the Frail status (subclassified as robust, pre-frail, and frail) after 6 months according to Fried's scale, and the quality of anesthesia assessed through the total duration in the expected interval of BIS value (40–60), the total duration in too deep anesthesia (defined as a BIS value <40), and the occurrence of a suppression ratio. The period of analysis is only that during general anesthesia. Based on the published data, it is estimated that at 6 months, the mortality in this population will be approximately 8%, institutionalization will be required in one in five of the remaining cases, and in the patients who remain alive without institutionalization, a decline in ADL will occur in one subject in six in 6 months. This leads to an overall proportion of patients who will die or whose level of functioning will deteriorate after a scheduled major surgical intervention of 39.4%. We hypothesize that better control of the depth of anesthesia should reduce this figure by one third. Under these conditions, a sample of 430 patients, equally divided between the two anesthesia groups, leads to a Fisher’s exact test with a risk of first species bilateral of 0.05 to a power of 0.8 to highlight the expected difference, if it exists.

This research will include a randomization of 1:1 between the groups with stratification based upon the frailty status of the subject prior to the surgery, as determined using the phenotypic Fried scale in three categories (robust, pre-frail, and frail) crossed with two surgical risks (minor or major) [19] (Table 1). The randomization list with balancing groups is performed using a computer program; allocation to a group is performed automatically.

**Table 1:** Determination of surgical risk (minor or major) in the elderly. (Adapted from Eagle [19])

Usual classification	Type of surgery	Adapted classification in elderly patients
Low Risk	Wall surgery without opening the peritoneal cavity	Low Risk
	Abscess drainage	
	Endoscopies	
	Proctology	
	Plastic surgery	
	Ophthalmic surgery	
Intermediate Risk	Intraperitoneal surgery	Major Risk
	Laparoscopic surgery	
	Endarterectomy	
	Ear, nose and throat (ENT) surgery	
	Prostate surgery	
	Orthopedic surgery	
	*Intermediate risk in elderly (>70 years old)*	
Major Risk	Intermediate surgery performed in emergency	
	Unstable hemodynamic conditions	
	Vascular surgery (aortic and peripheral) and thoracic surgery	
	Prolonged surgery (>4 h) with high fluid displacement or major bleeding	

A statistical intention-to-treat analysis will compare both groups (manual and automated general anesthesia) after 6 months through the generalized linear mixed model (GLIMM), which considers the type of anesthesia, frailty status, and surgery. A significant threshold at 0.05 is defined, and the software used for the analysis will be either R3.0 or SPSS20. A logistic regression or a multivariable analysis could be performed to establish some predictive factors if relevant. To ensure that the hypothesis was correct compared to observed data, an intermediary analysis is planned after 100 inclusions.

## Discussion

During the preoperative visit, elderly patients and their relatives are often more worried about undergoing general anesthesia, because of the risk of death or memory loss, than about the actual surgical procedure. We expect to demonstrate no impairment in the primary outcome in the automated control of anesthesia with accurate titration to maintain BIS in a tight range in comparison to an eventual relationship between manual control and a change in the postoperative status as it concerns mortality and self-sufficiency. This outcome was chosen because it is clinically relevant and represents a real healthcare challenge. Additionally, the role of anesthesiology remains unclear with regards to postoperative cognitive dysfunction if the depth of anesthesia is controlled. In parallel, this cohort may bring information about the course of the frailty status in the postoperative period. Can pre-frailty be a predictive factor for loss of self-sufficiency in the postoperative course?

The depth of general anesthesia is a common factor that may favor postoperative morbidity in the case of excessively deep sedation. This factor has been investigated in many retrospective studies that described a BIS value below 45 as a risk factor for postoperative death or organ failure [20]. This theory was recently reported as the “triple low” by Sessler et al. [21], i.e., low BIS, low arterial blood pressure, and low volatile agent concentration. However, few prospective studies have been designed to support or reject this hypothesis. Therefore, this randomized clinical trial may provide additional information for a specific population of elderly patients. To limit the variability of different brain susceptibilities to anesthetics in the elderly, each patient will be monitored using the BIS with a common target (BIS in the range 40–60), and anesthesia will be performed only with intravenous drugs to homogenize general anesthesia. Under these circumstances, we hypothesize that a significant decrease in the rate of patients with severe impairment in their autonomy, among patients receiving automated anesthesia compared to manually controlled anesthesia, will occur.

This hypothesis is supported by numerous studies. First, our group showed that automated anesthesia guided by the BIS sensor outperforms manual anesthesia regarding the duration in the expected range [22]. Therefore, this system seems capable of continuously adapting the posology of anesthetics depending on the immediate needs and eventual hemodynamic events. A sudden drop in cerebral output leads to a decrease in BIS and in site-effect concentrations, allowing a typical titration of drugs. This description was suggested in the case of cardiac arrest with a complete stop in infusion and a restart after recovery. This accurate adaptation to the patient’s needs is very important considering the literature on too deep sedation. The first description was reported in an intensive care unit with a dichotomy between a patient with burst suppression (isoelectric signal) and one without. In addition, an analysis of patients undergoing surgery with this kind of controller demonstrated a more frequent occurrence of isoelectric periods in the elderly without clear risk factors [7]. Different studies on severely ill patients have been performed using an automatic controller of anesthesia, with some publications on lung transplantations and patients in intensive care. Consequently, these results suggest a difference in brain susceptibility, as described with newborns.

Elderly patients comprise more than 40% of all surgical patients in the USA per year and generate a very large proportion of healthcare costs, especially in the postoperative period [23]. Studies suggest that frailty predisposes an individual to worsening health status and death and is likely unrecognized in elderly patients prior to surgery. Because relatively minor stressors (e.g., urinary tract infections) may result in major consequences such as increased dependence on caregivers, the need for nursing care, and predisposition to falls and delirium, major stressors such as surgery are likely related to a loss of autonomy and mortality in frail or pre-frail patients [24]. However, few studies have focused on the outcome of robust and pre-frail elderly patients compared to frail elderly ones as proposed in this trial. Once again, the concept of frailty transcends comorbidity alone to include strength, function, and cognition, and in many types of surgery, frailty status predicts postoperative complications better than traditional risk scores. This line of study is crucial for the elderly, especially for those with frailty symptoms. This study will provide information on the outcomes of pre-frail patients to determine if pre-frail patients may benefit from a pre-habilitation program to increase their physical function. This active program could help them become more robust and may lead to a better outcome.

This concept of patient optimization was initially described in patients with respiratory functions that limited surgery. Specifically, Gillis et al. in Montreal demonstrated a significant improvement in physical function after a 4-week program that included aerobic/anaerobic exercises, nutrition, and psychological support [25]. However, literature on pre-frail patients is scarce. Moreover, a complete psychometric examination aims to discriminate risk factors of cognitive dysfunction, loss of autonomy, and postoperative delirium. This examination could detect factors that should be investigated before a major intervention using a limited questionnaire. Indeed, the time devoted to presurgery in the elderly is limited in many countries, and additional tools are required to improve their postoperative care. Accordingly, this represents another objective for this large cohort of elderly patients. In a recent study, Revenig et al. demonstrated in 351 consecutive patients undergoing major intra-abdominal interventions that shrinking and grip strength alone hold the same prognostic information as the full five-component Fried frailty criteria for 30-day morbidity and mortality [5]. The addition of the ASA score and serum hemoglobin creates a composite risk score, which facilitates the classification of patients into discrete low, intermediate (odds ratio (OR) 1.974, 95% CI 1.006–3.877, *p* = 0.048), and high (OR 4.889, 95% CI 2.220–10.769, *p* < 0.001) risk categories, with a corresponding stepwise increase in risk for 30-day postoperative complications [5]. The use of muscle assessment is also a novel concept for patients with sarcopenia. Indeed, this loss in muscle mass and in performance largely impairs the daily life of patients, and some studies suggest a correlation between sarcopenia, which is measured through paravertebral muscle mass, and lumbar spine L3. This simple preoperative exam provides new information that should promote caution during the postoperative course [26].

The main limitation to our approach is the use of anesthesia in both groups using the BIS sensor, which is available at any moment for both groups. This could also lead to the optimization of the control group compared to the standard group. However, in the case of special morbidity as in elderly patients, this was the only way to make this comparison. Secondly, volatile anesthetics are not used in this study and may induce a different response. Thirdly, we have no knowledge of the rate of frail patients in the operating room, as surgeons can spontaneously reject an operation when faced with a patient with several comorbidities.

If our hypothesis is true, automated anesthesia could be recommended to maintain a similar frailty status among the elderly.

### Trial status

The inclusion of the patients and data started early in 2016. To date, the first 50 patients have been included and randomized and followed during at least 3 months. The patients’ pathway is now optimized to ensure follow-up and appropriate reporting of the data, and new centers will be opened shortly.

## Additional file

Additional file 1: SPIRIT 2013 checklist: recommended items to address in a clinical trial protocol and related documents. (DOC 123 kb)

## Abbreviations

ADL: Activities of daily living
ASA: American Society of Anesthesiologists
BIS: Bispectral index
EEG: Electroencephalogram
EKG: Electrocardiogram
IADL: Instrumental activities of daily living
OR: Odds ratio
